# Contamination Levels and Sources of Heavy Metals and a Metalloid in Surface Soils in the Kumasi Metropolis, Ghana

**DOI:** 10.5696/2156-9614-7.15.28

**Published:** 2017-09-07

**Authors:** Osei Akoto, Nesta Bortey-Sam, Yoshinori Ikenaka, Shouta M.M. Nakayama, Elvis Baidoo, Yared Beyene Yohannes, Mayumi Ishizuka

**Affiliations:** 1 Department of Chemistry, Kwame Nkrumah University of Science and Technology, Kumasi, Ghana; 2 Laboratory of Toxicology, Graduate School of Veterinary Medicine, Hokkaido University, Kita 18, Nishi 9, Kita ku, Sapporo 060-0818, Japan

**Keywords:** heavy metals, metalloid, Kumasi, contamination factor, pollution load index

## Abstract

**Background.:**

Environmental contamination with heavy metals and metalloids due to industrial, smelting and mining activities have become common in large and growing cities. Kumasi is one of the most industrialized cities in Ghana and experiences metal pollution due to recent and past activities. Although metals are naturally abundant in the area, their accumulation in soils could potentially lead to adverse effects on local ecosystems.

**Objectives.:**

The aims of this study were to determine the distribution, enrichment, geoaccumulation and sources of metals in Kumasi soils and to estimate the contamination factor (CF) and pollution load index (PLI) of these metals in soils.

**Methods.:**

Concentrations of eight heavy metals and a metalloid were determined in 112 soil samples randomly collected from 31 sampling sites in the area. In addition, 5 soil samples were collected from a pristine site (Kwame Nkrumah University of Science and Technology Botanical Gardens) for data comparison, to determine the local background values for metal concentrations and to evaluate the extent of metal pollution in the study area.

**Results.:**

Heavy metals such as zinc (Zn), lead (Pb), cadmium (Cd) and chromium (Cr) were enriched in 65, 32, 58 and 93% of the sampling sites, respectively, and geo-accumulation indexes for Cr, Zn, Cd, mercury (Hg) and Pb showed moderate to extreme contamination in 100, 97, 77, 65 and 45% of the sampling sites, respectively. Principal component and cluster analyses revealed that industrial activities including mining were the major sources of metals in Kumasi soils with high metal input in the community of Suame. Distribution maps revealed hotspots of Cd, nickel (Ni), arsenic (As), cobalt (Co), copper (Cu) and Pb in Suame. The highest CFs for Cu, Cd, Ni, As, Co and Pb highlighted anthropogenic inputs in Suame, while Hg was highest in Mbrom, Zn in Suntreso, and Cr in Aboabo.

**Conclusions.:**

The PLI of metals revealed Suame as the most polluted study site, while Anomangye and Bomso were the least polluted.

## Introduction

Contamination of soil with heavy metal and metalloids results mainly from anthropogenic activities including mining, smelting, tanning, draining of sewage and dumping of wastes. Although metals occur naturally in the environment, chemical and metallurgical industries are the most important sources of metal contamination.^1^ Heavy metals/metalloids cannot be easily degraded and are continuously being deposited into soil, water and sediment, causing pollution.^2^ Metal concentrations are greatest near towns, indicating their urban/industrial origins. Apart from destabilizing the ecosystem, accumulation of these toxic metals in the food web is a threat to public health and their potential long-term impact on the ecosystem cannot be ignored.^3^

Kumasi (6° 40′00″ N 1° 37′00″ W) is the capital city of the Ashanti Region and covers a land area of 254 km^2^ (98 sq miles). It is the second largest city in Ghana with over 2.5 million inhabitants. The land is dominated by middle Precambrian rocks and the major soil type is forest Ochrosols.^4^ On the basis of land use, the study area can be divided into a number of categories: agriculture, human settlement, vegetation cover, water bodies, and industrial. The human population and number of cars have drastically increased during the past few years. In addition, many gas/fuel stations, auto-mechanic/repair workshops, metals fabricators, tanning industries, mining operations (including illegal mining operations popularly known as galamsey), stone quarrying and sand mining industries are located in this region. These and many other anthropogenic factors have led to the release of heavy metals and metalloids into the environment.^4^,^5^

There have been a limited number of studies assessing the enrichment/pollution levels and sources of heavy metals and metalloids in the Kumasi metropolis. The objectives of the present study were to determine the concentrations of heavy metals and a metalloid in Kumasi soils, determine the levels of metal accumulation compared to a pristine site using enrichment factor and geo-accumulation index, develop distribution maps of heavy metals/metalloid throughout the sample site using geographic information system (GIS), identify the possible sources of metals by multivariate analysis, and evaluate the extent of metal pollution in Kumasi soils using contamination factor and pollution load index.

**Abbreviations**
*CF*: Contamination factor
*EF*: Enrichment factor
*GM*: Geometric mean
*I_geo_*: Geoaccumulation index
*KNUST*: Kwame Nkrumah University of Science and Technology
*PC*: Principal component
*PLI*: Pollution load index
*USEPA*: United States Environmental Protection Agency

## Methods

### Sampling

**Figure 1:**
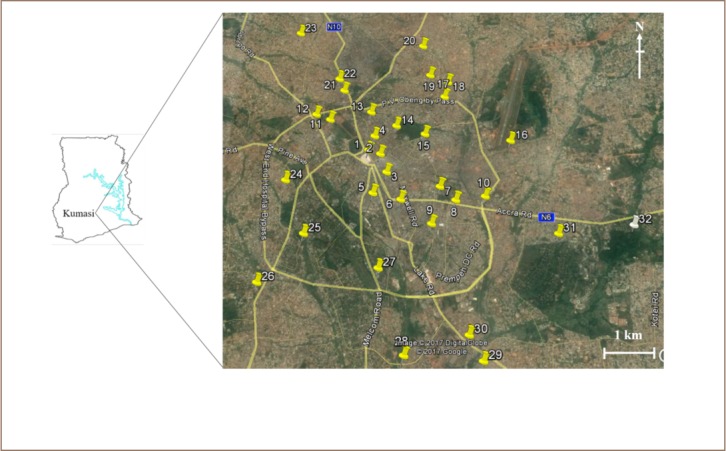
Sampling area/sites (yellow pins indicate sampled communities and white pin indicates reference site, KNUST Botanical Gardens). 1: Kejetia; 2: Central market; 3: Romanhill; 4: Mbrom; 5: Adum; 6: Asafo; 7: Amakom; 8: Afunkwanta; 9: Asokwa; 10: Oforikrom; 11: Racecourse; 12: Bantama; 13: Ashtown; 14: Manhyia; 15: Asawase; 16: Aboabo; 17: Dichemso; 18: Yennyawoso; 19: Tafo Nhyiaso; 20: Tafo; 21: New Suame; 22: Suame; 23: Anomangye; 24: Suntreso; 25: Danyame; 26: Patasi, 27: Ahodwo, 28: Kaase; 29: Atonsu; 30: Ahinsan; 31: Bomso and 32: KNUST Botanical Gardens.

Soil samples were randomly collected from 31 communities (sample sites) in the Kumasi metropolis. A map showing the sampling sites is presented in Figure 1. The sites were selected to represent a wide area of the town and global positioning system was used to locate the sampling positions. Sampling was done in May, 2011 and a total of 112 soils (0–10 cm top layer) were collected using a stainless steel scoop and stored in labeled Corning tubes (Corning Incorporated, New York, USA). In addition, due to the lack of known background soil concentrations in Ghana, 5 soil samples were collected from the Botanical Gardens of Kwame Nkrumah University of Science and Technology (KNUST) for data comparison (reference values), and to evaluate the extent of metal pollution and enrichment in the study samples. KNUST is a university located in Kumasi, Ghana, and because it experiences low vehicular traffic with no industrial (mining) activities, heavy metals from point sources were assumed to be negligible. Heavy metals and metalloid levels in KNUST Botanical Gardens soil were low compared to the world range for unpolluted soils.^6^ In addition, KNUST was used as a reference site for determination of polycyclic aromatic hydrocarbons in particulate matter and soils.^7–9^

All samples were stored at −20°C in the Department of Chemistry, KNUST, Ghana and later transported to the Laboratory of Toxicology, Graduate School of Veterinary Medicine, Hokkaido University, Japan, where they were stored at −30°C until analysis.

## Chemical Analysis

Prior to chemical analysis, soil samples were air-dried at room temperature and passed through a 2 mm sieve to remove the coarse soil fraction. Briefly, 1 g of dried soil was weighed into pre-washed digestion vessels and digested (Speed Wave MWS-2, Berghof, Germany) using 10.0 mL of 60% nitric acid, (atomic absorption spectrometry grade; Kanto Chemical Corporation, Tokyo, Japan). The microwave unit was calibrated to a temperature of 200°C and digestion was allowed for 45 minutes at 180 psi. After digestion, the solutions were allowed to cool, filtered using ashless filter paper 5B (Advantec, Tokyo, Japan) into Corning tubes (Corning Incorporated, New York, USA). Lanthanum chloride (1 mL, atomic absorption spectrometry grade, 100 g La/L solution, Wako Pure Chemical Industries Ltd., Osaka, Japan) was added to prevent ionization/interference during metal analysis. Samples were diluted to 50 mL with 2% nitric acid prepared with Milli-Q water. Concentrations of heavy metals and metalloids were determined by atomic absorption spectrophotometry (AAS) (Z-2010, Hitachi High Technologies Corporation, Tokyo, Japan) after preparation of calibration standards. Cadmium (Cd), chromium (Cr), nickel (Ni), lead (Pb) and arsenic (As) were analyzed by graphite furnace AAS (argon gas) with Zeeman background correction. Copper and zinc (Zn) were analyzed by flame AAS (acetylene flame) with deuterium background correction.

In addition, total mercury (Hg) was measured by thermal decomposition, gold amalgamation and atomic absorption spectrophotometry (mercury analyzer, MA–3000, Nippon Instruments Corporation, Tokyo, Japan), after preparation of calibration standards. Blanks were prepared using the same procedure.

The water content (WC) of each sample was measured after 12 h of oven drying at 105°C. Organic matter (OM) content was determined by loss of weight on ignition at an oven temperature of 600°C for 5 hours. Then pH was measured in a soil deionized water suspension (soil: water, 1:2.5 by volume) with a calibrated pH meter.

### Quality Control and Quality Assurance

For quality control, blanks were analyzed after every 10 samples. The instrument was calibrated using standard solutions of the respective metals (to establish standard curves before metal analysis). The detection limits (mg/kg) were 0.5 for Cr, 0.5 for cobalt (Co), 1.0 for copper (Cu), 0.1 for Zn, 0.2 for Cd, 1.0 for Pb, 0.5 for Ni and 2.0 for As. Reference materials, Standard Reference Material 1944 (New York/New Jersey Waterway Sediment) and BCR–320 (Channel Sediment, Institute for Reference Materials and Measurements, Belgium) were used for method validation. Replicate analyses of these reference materials showed good accuracy and recovery rates ranged from 80% to 115%. Recovery rates (%) of Hg for these certified reference materials (BCR–320R and Standard Reference Material 1944) ranged from 92–103. The detection limit of Hg in soil samples was 2.0 pg total Hg.

### Data Analysis

#### Enrichment Factor

A common approach to estimating the anthropogenic impact on soil is to calculate the enrichment factor (EF) for metal concentrations above uncontaminated background levels. The EF method normalizes the measured metal content with respect to a reference metal.^10^ The reference material would then represent a benchmark to which the metal concentrations in the polluted samples are compared and measured. Pollution, in this case, will be measured as the amount (or ratio) of the sample metal enrichment above the concentrations present in the reference sample. Similar to the approach by Matthai and Birch, Co was considered a normalizing element for determining anthropogenic pollution sources in this study.^11^ The EF was calculated according to Equation 1:

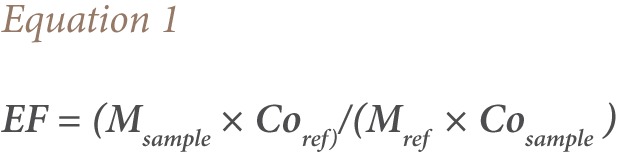
where M_sample_ is the geometric mean (GM) concentration of metal in soil, Co_ref_ is the GM of Co in the reference sample (KNUST Botanical Gardens), Mref is the GM of metal in reference sample and Co_sample_ is the GM of Co in the sample. Classification of EF by Chen et al. was adopted in this study and has been provided in Supplemental Material 1.^12^

#### Geoaccumulation Index

This method assesses the degree of metal pollution in terms of seven enrichment classes based on the increasing numerical values of the index. Geoaccumulation index (I_geo_) is calculated using Equation 2:

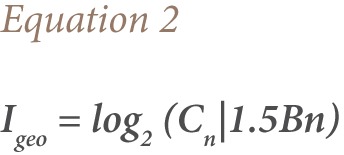
Where C_n_ is the GM concentration of the element in the enriched samples, and Bn is the background or pristine value of the element (KNUST Botanical Gardens). A factor of 1.5 was introduced to minimize the effect of possible variations in the background values which may be attributed to lithologic variations.^13^ Classification of I_geo_ by Muller was used (*Supplemental Material 1*).^14^

#### Contamination Factor

Contamination factor (CF) reflects the anthropogenic input in elemental pollution and is widely used as a measure of overall contamination of soil. CF was calculated using Equation 3:

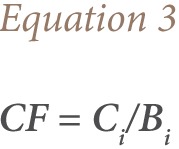
Where C_i_ and B_i_ are GM concentrations of the examined metal, i, in sample and reference (KNUST Botanical Gardens), respectively.^15^ The contamination grades in an increasing order are rated from 1 to 6 (0 = none, 1 = none to medium, 2 = moderate, 3 = moderate to strong, 4 = strongly polluted, 5 = strong to very strong, 6 = very strong).^16^

#### Pollution Load Index

The pollution load index (PLI) for a set of “n” polluting elements is defined as a value calculated from the GM of the contamination factors of those elements. PLI was calculated by the following expression given by Tomlinson et al: 17

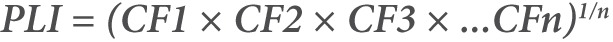
A PLI value higher than unity suggests pollution, while values lower than 1 indicate no pollution load.

### Statistical Analysis

Statistical analyses were performed using SPSS 20.0 (IBM SPSS Inc., Chicago, USA). Kolmogorov–Smirnov and Shapiro–Wilk's tests were used to determine the normality of data and data were considered statistically significant if the *p* value was less than 0.05. Statistical analyses were carried out after data were log transformed (normalized). Spatial distributions were performed using Arc-GIS 9.3 (ESRI Co., Redlands, USA). Kriging was adopted for the interpolation of geographical data. Geographic Information System coordinates and concentrations of metals obtained in soil were used to create the distribution maps. In order to identify the important parameters that affect the chemistry of soil, Pearson's correlation matrix was used (at a significance level (p) less than 0.05). Principal component analysis and cluster analysis were carried out to describe the degree of association/possible sources of metals in Kumasi soils. The principal components based on log transformed data were extracted with eigenvalues > 1 through a varimax rotation. Cluster analysis was also performed based on Euclidean distance using Ward's clustering method.

## Results

### Heavy Metals and Metalloid Concentrations in Kumasi Soils

Concentrations of eight heavy metals and a metalloid (As) were measured in Kumasi soils and results are shown in Table 1. The metal concentrations from some sample sites exceeded the recommended levels by Kabata-Pendias and Pendias (unpolluted soils), the United States Environmental Protection Agency (USEPA) and the reference site (KNUST Botanical Gardens).^6^,^18^ Kolmogorov-Smirnov tests for normality showed a significant variation (*p* < 0.01) in metal distribution in Kumasi soils (*Table 1*). As shown in Table 1, Zn was the most abundant metal (107 ± 64.6 mg/kg dry weight (dw)), followed by Cr (97.0 ± 43.7 mg/kg dw) and Pb (52.8 ± 37.9 mg/kg dw). Concentrations of Hg (0.05 ± 0.0363 mg/kg dw) and Cd (0.147 ± 0.159 mg/kg dw) were the lowest.

**Table 1:**
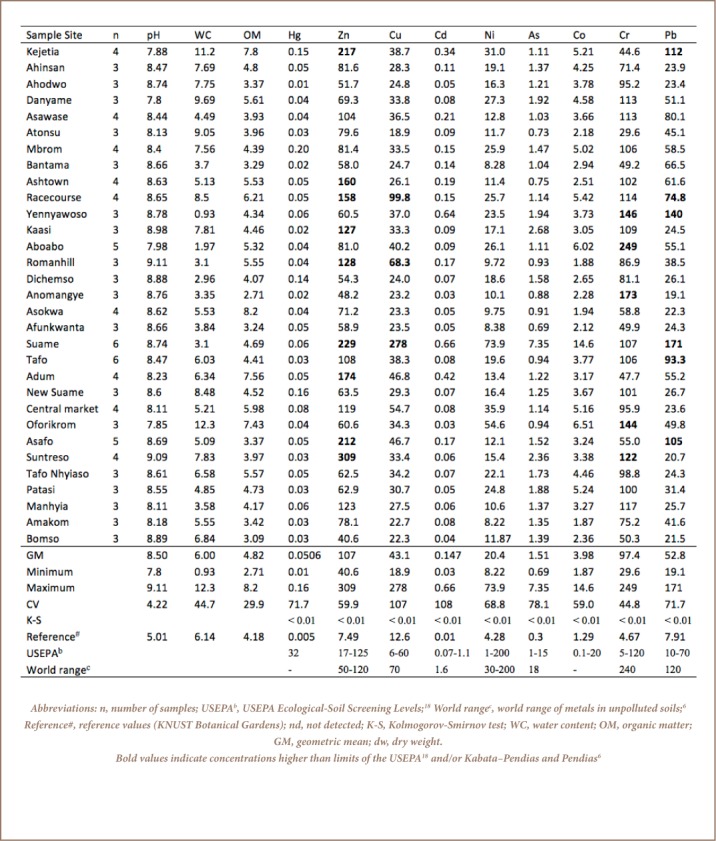
Geometric Mean Concentrations (mg/kg dw) of Heavy Metals and Metalloid in Soils in Kumasi, Ghana

### Enrichment Factor and Geoaccumulation Index

The results of EF revealed that 65, 32, 58 and 93% of soils from the sample sites were moderately to extremely high enriched with Zn, Pb, Cd and Cr, respectively (*Supplemental Material 2*), suggesting anthropogenic inputs. As shown in Table 2, Yennyawoso and Anomangye were extremely highly enriched with Cd (24.6) and Cr (21.1), respectively, and revealed extreme contamination (*I*_geo_; *Supplemental Material 3*) in these areas. The I_geo_ values for Cr, Zn, Cd, Hg and Pb indicated moderate to extreme contamination (I_geo_ classes 3 to 6) in 100, 97, 77, 65 and 45% of the sample sites, respectively, (*Supplemental Material 3*) when compared the reference site. In Suame, all the studied metals showed moderate to extreme contamination with I_geo_ ranging from 2.92 (Co) to 5.62 (Cd) (*Supplemental Material 3*).

**Table 2:**
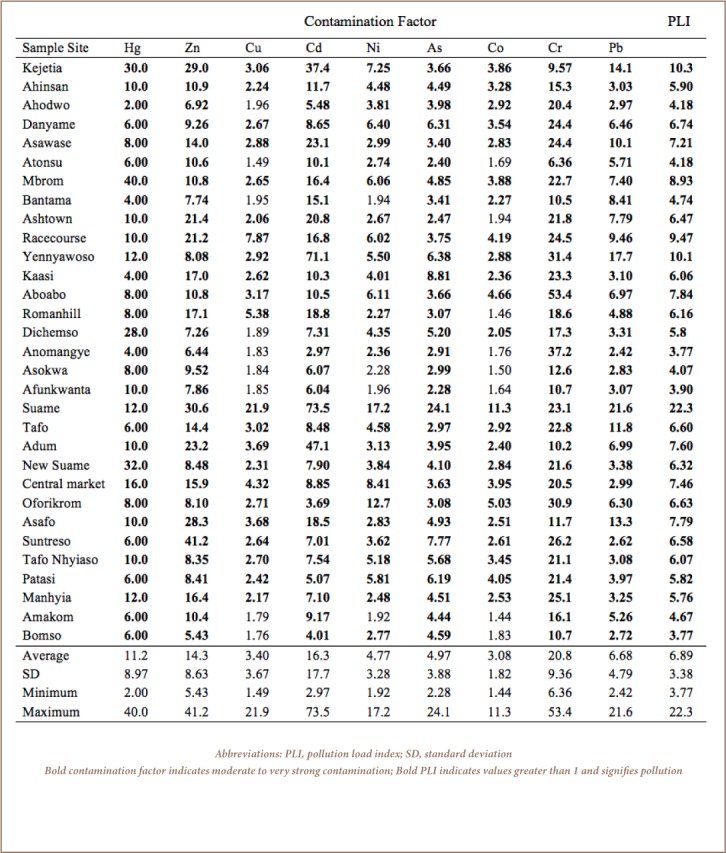
Contamination Factor and Pollution Load Index of Heavy Metals in Soils in Kumasi, Ghana

### Sources Characterization by Multivariate Analysis

#### Principal Component Analysis

**Figure 2:**
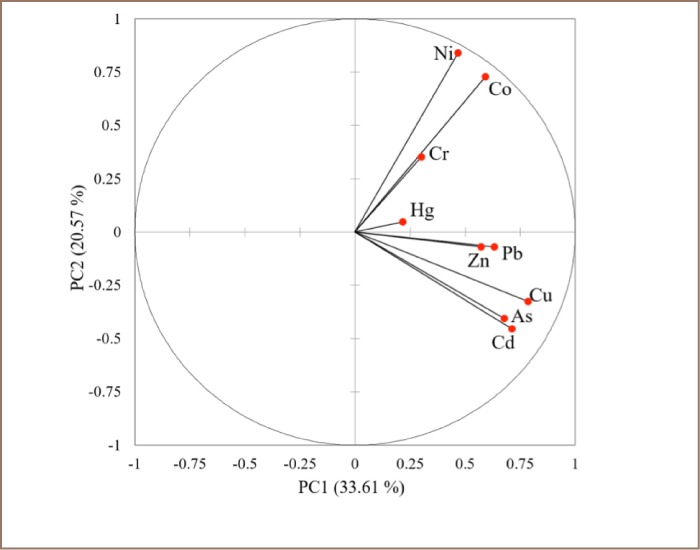
Source characterization of heavy metals and metalloid concentrations by principal component analysis

Principal component (PC) analysis was used to characterize sources of metals in Kumasi soils. From the results, three principal components (PC1, PC2, and PC3) were extracted and accounted for 66.9% of the total variance. As shown in Figure 2, PC1 explained 33.6% of the total variance and was characterized by high loadings of Cu, Pb, Cd, As and Zn. PC2 explained 20.6% of the total variance (*Figure 2*) and was dominated by high loadings of Ni, Co, and Cr, while PC3 (Hg) explained 12.7% of the total variance.

#### Cluster Analysis

**Figure 3:**
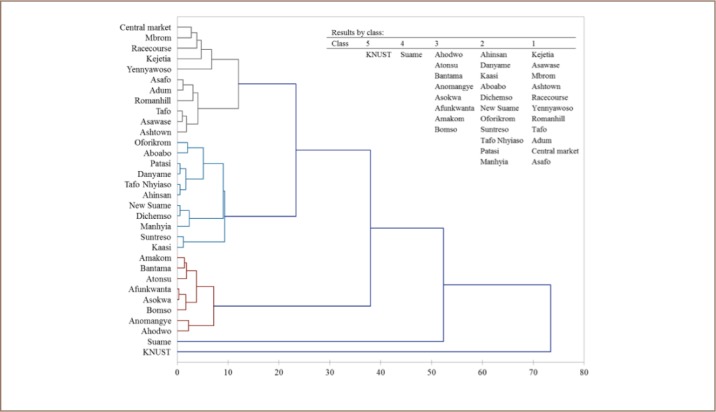
Hierarchical dendrogram of heavy metals and metalloid concentrations from the sample sites obtained using Ward's clustering method

Hierarchical cluster analysis was used in this study to identify the degree of association and/or accumulation pattern of heavy metals from the sample sites (*Figure 3*). The dendrogram revealed five classes with Suame and KNUST Botanical Gardens in different clusters (*Figure 3*). Kejetia, Asawase, Mbrom, Ashtown, Racecourse, Yennyawoso, Romanhill, Tafo, Adum, Central market and Asafo were in class 1 of the dendrogram (*Figure 3*), indicating a strong association between metal levels, sources or distribution in soils at these sites.

### Correlation Between Metals and Soil Properties

The physico-chemical parameters determined in Kumasi soils were soil pH, organic matter and water content. Mean pH values ranged from 7.88 ± 0.49 (Kejetia) to 9.11 ± 0.5 (Romanhill) (*Table 1*). pH influences the rate of adsorption, retention and the transfer/migration of heavy metals in soil.^19^ The organic matter content ranged from 2.71 (Anomangye) to 8.2% (Asokwa) (*Table 1*). In the present study, concentrations of metals did not correlate with either pH or organic matter (*Supplemental Material 4*), similar to the results of a previous study.^20^

### Distribution Maps of Heavy Metals and a Metalloid

**Figure 4:**
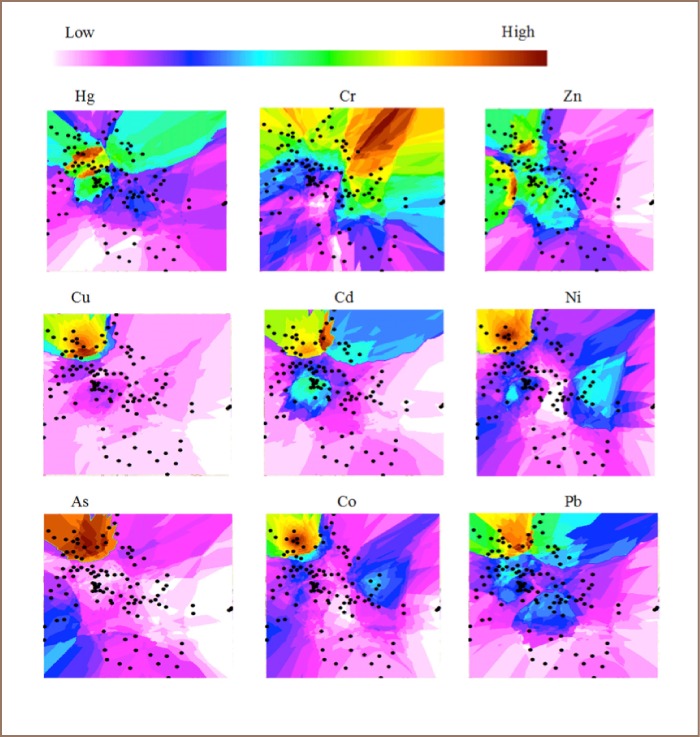
Distribution maps of metals in Kumasi soils, Ghana

The distribution maps of Cd, Ni, As, Co, Cu and Pb highlighted Suame (*22 on Figure 1*) as hotspot zone, indicating high metal concentrations (*Figures 1 and 4*). The hotspots identified from the geochemical map for Zn, Cr and Hg indicated high concentrations of these metals in Suntreso, Aboabo/Yennyawoso and Mbrom/Kejetia/New Suame, respectively.

### Contamination Factor and Pollution Load Index

Table 2 shows the CF and PLI of metals in soils from the sample sites. The average CF for all the studied metals were greater than 3, indicating moderate to very strong contamination in Kumasi soils. The highest CF for Cu, Cd, Ni, As, Co and Pb were in Suame, while the highest CF for Hg was in Mbrom, Zn (Suntreso), and Cr in Aboabo (*Table 2)*. In this study, the PLI of heavy metals and a metalloid revealed Suame (22.3) as the sample site most polluted with metals, followed by Kejetia (10.3) ≥ Yennyawoso (10.1) > Racecourse (9.47) > Mbrom (8.93) > Aboabo (7.84) ≥ Asafo (7.79) ≥ Adum (7.60) ≥ Central market (7.46) > …, Afunkwanta (3.90) ≥ Bomso (3.77) = Anomangye (3.77). The study showed that all study sites (average PLI = 6.89 ± 3.38; range: 3.77 to 22.3) were polluted with metals when compared to KNUST Botanical Gardens (*Table 2*).

## Discussion

### Metal Enrichment and Accumulation in Kumasi Soils

The concentrations, EF and I_geo_ of heavy metals and a metalloid from some sample sites within the Kumasi metropolis were higher and this could be due to the fact that the sampling sites in Kumasi were located within an area with heavy vehicular movement/traffic and industrialization. In addition, the high soil metal enrichment could be attributed to differences in the magnitude of input for each metal and/or differences in the removal rate from soil.^5^ The moderate to extreme metal contamination in Suame could have resulted from the many auto mechanical/repair workshops and vehicles in the area.

### Principal Component Analysis

As shown in Figure 2, PC1 showed an association with Cu, Pb, Cd, As and Zn and inputs of these metals and metalloid could have resulted from industrial activities and discharges such as mining and smelting processes.^21^ Cd is soft, ductile and is obtained as a by-product from the smelting of Zn ores. In mining contexts, Cd can also be found in the form of the greenockite mineral, cadmium sulfide. Cadmium in soils from the study area may come from the mining and processing of Zn and chalcophilic metals.^22^ The presence of Zn in the environment is associated with mining and smelting, which pollutes the air, water and soil and ultimately undergoes oxidation to release Zn^2+^ ions into water bodies.^22^

Processing ore after blasting gold bearing rock involves roasting, and this results in the production of arsenic trioxide gas which is distributed throughout the study area by air currents. Arsenic is toxic and could accumulate in surface soils because of its non-biodegradable nature.^23^ Cadmium and As concentrations could also be associated with industrial activities/discharges, sewage sludge and municipal solid waste.^24^

Large amounts of vehicular/industrial emissions and improper disposal of wastes could have contributed to the Cu, Pb, and Zn levels in Kumasi. Although no longer in use, previous use of leaded fuel could have accounted for the levels of Pb in soils. Lead is considered immobile in sub-surface soil and generally gets accumulated and remains strongly bound to soil mineral or organic components when deposited.^25^

Ni, Co and Cr were grouped in the same principal component. The high EF and I_geo_ from the sample sites, especially for Cr, suggest an anthropogenic input from industrial sources.^26^ Ghana, including the Kumasi metropolis, is filled with many tanning industries where animal skin is converted into leather, a practice considered to be an environmental threat. During this process, a variety of chemicals are used along with large volumes of water which are discharged as effluents containing liquid and solid wastes, and a significant amount of Cr.^27^ An average tannery uses approximately 65,000 tons of chemicals, including Cr (as chromium sulfate), annually, only to be discharged as effluents.^28^ The processes involved in the treatment of skin/hides are highly associated with sodium, potassium, magnesium and Co in effluents. Similarly, tanning processes are highly associated with Cr in soil.^27^

Artisanal and small-scale gold mining, production of cement and non-ferrous metals (including Cu, Pb, Zn, aluminum and large-scale gold production) and disposal of Hg wastes are some of the main sources of environmental Hg contamination.^29^ Artisanal and small-scale mining is highly practiced in some sample sites in Kumasi, and during this process Hg is used to amalgamate gold.^23^,^30^ This practice, although simple and inexpensive, could contribute to the concentrations of metals, including Hg levels, in the environment. Other sources of Hg in Kumasi soils could include discarded thermometers, batteries and fluorescent lamps, as these accounted for 40% of Hg emissions in North America. In addition, the use of barometers releases Hg into the environment.^31^ In agricultural systems, pesticides, fertilizers, sewage sludge and irrigation water were some of the main sources of Hg contamination.^32^

### Cluster Analysis

KNUST Botanical Gardens (reference site) was grouped in a different cluster of the dendrogram (*Figure 3*) and this trend could be attributed to the low metal concentrations compared to other sites and the world range for unpolluted soils.^6^ This trend, again, suggests that metals detected in KNUST Botanical Garden soils originated from natural/geological processes. On the other hand, high levels of metals were detected in Suame (*Table 1*) which was in class 4 of the dendrogram (*Figure 3*) and, EF and I_geo_ indicated moderate to extreme contamination at this site (*Supplemental Material 1 and 3*). Differences in metal sources or the higher level of soil metal contamination could have contributed significantly to the present results.

Sampling sites such as Kejetia, Asawase, Mbrom, Ashtown, Racecourse, Yennyawoso, Romanhill, Tafo, Adum, Central market and Asafo were grouped in class 1 of the dendrogram (*Figure 3*), implying a similar/single source of metal pollution at these sites. Sources of metals in Mbrom and Central market, and Adum and Asafo were closely related and showed a strong association with metals levels in Racecourse, Kejetia and Yennyawoso (*Figure 3*). As shown in Figure 1, the sampling sites grouped in class 1 are close together, with the exception of Yennyawoso (site # 18) and Tafo (site # 20), which are about 1.3 km apart. This trend could possibly be due to effects of artisanal and small-scale gold mining in some sample sites.

### Correlation Between Metals and Soil Properties

Lack of significant correlation between soil properties and heavy metals could be attributed to a continuous input, since the release and transport of heavy metals are governed by complex processes.^33^,^34^ Another possible reason could be variations in soil type within the sampled area.^33^,^34^

### Distribution Maps of Heavy Metals and a Metalloid

As shown in the distribution maps (*Figure 4*), the high metal concentrations in Suame could also be due to heavy vehicle traffic and the large amount of metal scrap in the area. The “Suame Magazine” is one of Africa's largest light-industrial clusters. It is a 200 hectare area filled with polluted industrial waste, auto-mechanic workshops and saw mills, with frequent burning of tires generating a significant amount of waste.^35^ The hotspots identified from the distribution maps for Zn (Suntreso), Cr (Aboabo and Yennyawoso) and Hg (Mbrom, Kejetia and New Suame) could be due to the fact that some of these sampling sites are densely populated with vehicles and light (small scale) industries, including many tanneries.

## Conclusions

Concentrations of heavy metals and a metalloid were studied in Kumasi soils and Zn, Pb, Cd and Cr were found to be enriched in some of the sample sites, suggesting anthropogenic inputs. Based on the obtained I_geo_ values, Cr, Zn, Cd, Hg and Pb showed moderate to extreme contamination in 100, 97, 77, 65 and 45% of the sample sites, respectively. Distribution maps and pollution load index values highlighted Suame as the most significantly polluted site, followed by Kejetia ≥ Yennyawoso > Racecourse > Mbrom > Aboabo ≥ Asafo ≥ Adum ≥ Central market > …, Afunkwanta ≥ Bomso = Anomangye. Finally, the present study indicates that industrial activities, including mining, are the major sources of metals in Kumasi soils, as observed at the most contaminated site of Suame.
