# Supplementary material for: Contamination Levels and Sources of Heavy Metals and a Metalloid in Surface Soils in the Kumasi Metropolis, Ghana
Source: J Health Pollut. 2017 Sep 7;7(15):28–39. doi: 10.5696/2156-9614-7.15.28 (PMC6236539; doi:10.5696/2156-9614-7.15.28)
Supplement: Supplementary file 1 [file Akoto_Supplemental.docx]

**Supplemental Material 1**

**Designation of Enrichment Factor^12^ and Geoaccumulation Index Values^14^**

| EF Range | Enrichment Grade | Enrichment Level | I_geo_ Value | I_geo_ Class | Designation of Sediment Quality |
| --- | --- | --- | --- | --- | --- |
| EF < 1 | 1 | without enrichment | 0 | 0 | uncontaminated |
| 1 ≤ EF < 3 | 2 | deficiency to minimal enrichment | 0–1 | 1 | uncontaminated to moderately contaminated |
| 3 ≤ EF < 5 | 3 | moderate enrichment | 1–2 | 2 | moderately contaminated |
| 5 ≤ EF < 10 | 4 | moderate to high enrichment | 2–3 | 3 | moderately to strongly contaminated |
| 10 ≤ EF < 25 | 5 | high enrichment | 3–4 | 4 | strongly contaminated |
| 25 ≤ EF < 50 | 6 | very high enrichment | 4–5 | 5 | strongly to extremely contaminated |
| 50 ≤ EF | 7 | extremely high enrichment | > 5 | 6 | extremely contaminated |

Abbreviations: EF, enrichment factor; I_geo_, geoaccumulation index

**Supplemental Material 2**

**Enrichment Factor Values for Soil Samples in Kumasi, Ghana**

| Sample site | Hg | Zn | Cu | Cd | Ni | As | Co | Cr | Pb |
| --- | --- | --- | --- | --- | --- | --- | --- | --- | --- |
| Kejetia | 1.22 | **7.51** | 0.79 | **9.69** | 1.88 | 0.09 | 1.00 | 2.48 | **3.67** |
| Ahinsan | 0.43 | **3.32** | 0.68 | **3.58** | 1.37 | 0.14 | 1.00 | **4.66** | 0.92 |
| Ahodwo | 0.15 | 2.37 | 0.67 | 1.88 | 1.31 | 0.14 | 1.00 | **6.98** | 1.02 |
| Danyame | 0.27 | 2.62 | 0.75 | 2.44 | 1.81 | 0.18 | 1.00 | **6.89** | 1.82 |
| Asawase | 0.38 | **4.95** | 1.02 | **8.16** | 1.06 | 0.12 | 1.00 | **8.62** | **3.58** |
| Atonsu | 0.54 | **6.30** | 0.88 | **6.03** | 1.62 | 0.14 | 1.00 | **3.77** | **3.38** |
| Mbrom | 1.56 | 2.80 | 0.68 | **4.24** | 1.56 | 0.12 | 1.00 | **5.85** | 1.90 |
| Bantama | 0.30 | **3.41** | 0.86 | **6.68** | 0.85 | 0.15 | 1.00 | **4.64** | **3.70** |
| Ashtown | 0.67 | **11.1** | 1.06 | **10.7** | 1.38 | 0.13 | 1.00 | **11.2** | **4.02** |
| Racecourse | 0.34 | **5.06** | 1.88 | **4.02** | 1.44 | 0.09 | 1.00 | **5.87** | 2.26 |
| Yennyawoso | 0.54 | 2.80 | 1.01 | **24.6** | 1.91 | 0.22 | 1.00 | **10.8** | **6.15** |
| Kaasi | 0.20 | **7.21** | 1.11 | **4.37** | 1.70 | 0.37 | 1.00 | **9.89** | 1.31 |
| Aboabo | 0.24 | 2.32 | 0.68 | 2.27 | 1.31 | 0.08 | 1.00 | **11.4** | 1.50 |
| Romanhill | 0.65 | **11.7** | **3.70** | **12.9** | 1.56 | 0.21 | 1.00 | **12.7** | **3.35** |
| Dichemso | 1.95 | **3.54** | 0.92 | **3.56** | 2.12 | 0.25 | 1.00 | **8.46** | 1.61 |
| Anomangye | 0.26 | **3.66** | 1.04 | 1.69 | 1.34 | 0.17 | 1.00 | **21.1** | 1.38 |
| Asokwa | 0.67 | **6.34** | 1.23 | **4.04** | 1.52 | 0.20 | 1.00 | **8.39** | 1.88 |
| Afunkwanta | 0.90 | **4.80** | 1.13 | **3.69** | 1.20 | 0.14 | 1.00 | **6.53** | 1.88 |
| Suame | 0.14 | 2.70 | 1.94 | **6.48** | 1.52 | 0.21 | 1.00 | 2.03 | 1.91 |
| Tafo | 0.31 | **4.96** | 1.04 | 2.91 | 1.57 | 0.10 | 1.00 | **7.84** | **4.05** |
| Adum | 0.61 | **9.70** | 1.54 | **19.6** | 1.31 | 0.16 | 1.00 | **4.26** | 2.92 |
| New Suame | 1.71 | 2.99 | 0.81 | 2.79 | 1.36 | 0.14 | 1.00 | **7.64** | 1.19 |
| Central market | 0.58 | **4.05** | 1.09 | 2.24 | 2.13 | 0.09 | 1.00 | **5.21** | 0.76 |
| Oforikrom | 0.27 | 1.61 | 0.54 | 0.73 | 2.54 | 0.06 | 1.00 | **6.15** | 1.25 |
| Asafo | 0.62 | **11.3** | 1.47 | **7.40** | 1.13 | 0.20 | 1.00 | **4.71** | **5.31** |
| Suntreso | 0.38 | **15.8** | 1.01 | 2.68 | 1.39 | 0.30 | 1.00 | **10.0** | 1.00 |
| Tafo Nhyiaso | 0.44 | 2.42 | 0.78 | 2.18 | 1.50 | 0.16 | 1.00 | **6.13** | 0.89 |
| Patasi | 0.24 | 2.07 | 0.60 | 1.25 | 1.43 | 0.15 | 1.00 | **5.30** | 0.98 |
| Manhyia | 0.72 | **6.50** | 0.86 | 2.81 | 0.98 | 0.18 | 1.00 | **9.94** | 1.28 |
| Amakom | 0.53 | **7.23** | 1.24 | **6.35** | 1.33 | 0.31 | 1.00 | **11.1** | **3.64** |
| Bomso | 0.50 | 2.97 | 0.96 | 2.19 | 1.52 | 0.25 | 1.00 | **5.90** | 1.49 |
| KNUST Botanical Gardens | 1.00 | 1.00 | 1.00 | 1.00 | 1.00 | 1.00 | 1.00 | 1.00 | 1.00 |

Bold indicates moderate to extremely high enrichment

**Supplemental Material 3**

**Geoaccumulation Index for Metals in Soil Samples in Kumasi, Ghana**

| Igeo | Hg | Zn | Cu | Cd | Ni | As | Co | Cr | Pb |
| --- | --- | --- | --- | --- | --- | --- | --- | --- | --- |
| Kejetia | **4.32** | **4.27** | 1.03 | **4.64** | **2.27** | 1.29 | 1.37 | **2.67** | **3.24** |
| Ahinsan | **2.74** | **2.86** | 0.58 | **2.97** | 1.58 | 1.58 | 1.13 | **3.35** | 1.01 |
| Ahodwo | 0.42 | **2.20** | 0.38 | 1.87 | 1.35 | 1.41 | 0.96 | **3.77** | 0.98 |
| Danyame | 2.00 | **2.63** | 0.83 | **2.53** | **2.09** | **2.07** | 1.24 | **4.02** | **2.11** |
| Asawase | **2.42** | **3.22** | 0.94 | **3.95** | 1.00 | 1.18 | 0.92 | **4.02** | **2.76** |
| Atonsu | 2.00 | **2.83** | -0.01 | **2.76** | 0.87 | 0.68 | 0.17 | **2.08** | 1.93 |
| Mbrom | **4.74** | **2.86** | 0.82 | **3.46** | 2.02 | 1.69 | 1.37 | **3.92** | **2.30** |
| Bantama | 1.42 | **2.37** | 0.38 | **3.34** | 0.37 | 1.18 | 0.60 | **2.81** | **2.49** |
| Ashtown | **2.74** | **3.84** | 0.46 | **3.80** | 0.83 | 0.72 | 0.37 | **3.87** | **2.38** |
| Racecourse | **2.74** | **3.82** | **2.39** | **3.49** | 2.01 | 1.32 | 1.48 | **4.03** | **2.66** |
| Yennyawoso | **3.00** | **2.43** | 0.96 | **5.57** | 1.88 | **2.09** | 0.94 | **4.39** | **3.56** |
| Kaasi | 1.42 | **3.50** | 0.81 | **2.78** | 1.42 | **2.55** | 0.66 | **3.96** | 1.05 |
| Aboabo | **2.42** | **2.85** | 1.08 | **2.81** | 2.03 | 1.29 | 1.63 | **5.15** | **2.22** |
| Romanhill | **2.42** | **3.52** | 1.84 | **3.65** | 0.60 | 1.03 | -0.04 | **3.63** | 1.70 |
| Dichemso | **4.22** | **2.27** | 0.34 | **2.29** | 1.54 | 1.79 | 0.45 | **3.53** | 1.14 |
| Anomangye | 1.42 | **2.10** | 0.29 | 0.98 | 0.65 | 0.95 | 0.23 | **4.63** | 0.69 |
| Asokwa | **2.42** | **2.67** | 0.30 | 2.02 | 0.60 | 0.99 | 0.00 | **3.07** | 0.91 |
| Afunkwanta | **2.74** | **2.39** | 0.30 | 2.01 | 0.38 | 0.60 | 0.13 | **2.83** | 1.03 |
| Suame | **3.00** | **4.35** | **3.87** | **5.62** | **3.53** | **4.01** | **2.92** | **3.94** | **3.85** |
| Tafo | 2.00 | **3.27** | 1.01 | **2.50** | 1.61 | 0.98 | 0.96 | **3.93** | **2.98** |
| Adum | **2.74** | **3.95** | 1.30 | **4.97** | 1.06 | 1.39 | 0.68 | **2.77** | **2.22** |
| New Suame | **4.42** | **2.50** | 0.62 | **2.40** | 1.36 | 1.45 | 0.92 | **3.85** | 1.17 |
| Central market | **3.42** | **3.41** | 1.52 | **2.56** | **2.49** | 1.27 | 1.40 | **3.78** | 1.00 |
| Oforikrom | **2.42** | **2.43** | 0.85 | 1.30 | **3.09** | 1.04 | 1.74 | **4.37** | **2.07** |
| Asafo | **2.74** | **4.24** | 1.30 | **3.63** | 0.92 | 1.71 | 0.74 | **2.98** | **3.15** |
| Suntreso | 2.00 | **4.78** | 0.82 | **2.22** | 1.27 | **2.37** | 0.80 | **4.13** | 0.81 |
| Tafo Nhyiaso | **2.74** | **2.48** | 0.85 | **2.33** | 1.79 | 1.92 | 1.20 | **3.82** | 1.04 |
| Patasi | 2.00 | **2.49** | 0.69 | 1.76 | 1.95 | 2.04 | 1.43 | **3.84** | 1.40 |
| Manhyia | **3.00** | **3.45** | 0.53 | **2.24** | 0.72 | 1.59 | 0.75 | **4.07** | 1.11 |
| Amakom | 2.00 | **2.80** | 0.26 | **2.61** | 0.36 | 1.56 | -0.05 | **3.43** | 1.81 |
| Bomso | 2.00 | 1.86 | 0.23 | 1.42 | 0.89 | 1.61 | 0.29 | **2.85** | 0.86 |
| KNUST Botanical Garden | -0.58 | -0.58 | -0.58 | -0.58 | -0.58 | -0.58 | -0.58 | -0.58 | -0.58 |

Abbreviation: I_geo_, geoacumulation index
Bold indicates moderate to extremely contaminated

**Supplemental Material 4**

**Pearson Correlation Between Metals, Organic Matter and pH**

|  | Hg | Cu | Zn | Pb | Cd | Co | Ni | As | Cr | OM | pH |
| --- | --- | --- | --- | --- | --- | --- | --- | --- | --- | --- | --- |
| Hg | 1.00 |  |  |  |  |  |  |  |  |  |  |
| Cu | -0.14 | 1.00 |  |  |  |  |  |  |  |  |  |
| Zn | -0.04 | 0.24 | 1.00 |  |  |  |  |  |  |  |  |
| Pb | -0.16 | **0.51** | 0.24 | 1.00 |  |  |  |  |  |  |  |
| Cd | -0.08 | 0.37 | 0.09 | 0.15 | 1.00 |  |  |  |  |  |  |
| Co | -0.08 | 0.01 | 0.24 | 0.01 | -0.02 | 1.00 |  |  |  |  |  |
| Ni | -0.10 | -0.09 | -0.05 | 0.02 | -0.18 | **0.90** | 1.00 |  |  |  |  |
| As | -0.11 | **0.41** | 0.00 | -0.11 | **0.69** | 0.03 | -0.18 | 1.00 |  |  |  |
| Cr | -0.19 | -0.08 | -0.04 | -0.08 | -0.16 | 0.08 | 0.08 | -0.11 | 1.00 |  |  |
| OM | 0.07 | -0.02 | 0.01 | -0.16 | 0.00 | -0.10 | -0.05 | -0.04 | -0.09 | 1.00 |  |
| pH | -0.38 | -0.15 | -0.19 | -0.17 | -0.15 | -0.17 | -0.22 | -0.19 | -0.15 | -0.37 | 1.00 |

Abbreviations: OM, organic matter

Bold values indicate significance at *p* < 0
